# Therapeutic Potential of PPAR*γ* Activation in Stroke

**DOI:** 10.1155/2008/461981

**Published:** 2008-04-13

**Authors:** Raghu Vemuganti

**Affiliations:** Department of Neurological Surgery and the Neuroscience Training Program, University of Wisconsin-Madison, Madison, WI 53792, USA

## Abstract

Stroke (focal cerebral ischemia) is a leading cause of death and disability among adult population. Many pathological events including inflammation and oxidative stress during the acute period contributes to the secondary neuronal death leading the neurological dysfunction after stroke. Transcriptional regulation of genes that promote these pathophysiological mechanisms can be an effective strategy to minimize the poststroke neuronal death. Peroxisome proliferator-activated receptors (PPARs) are ligand-activated transcription factors known to be upstream to many inflammatory and antioxidant genes. The goal of this review is to discuss the therapeutic potential and putative mechanisms of neuroprotection following PPAR activation after stroke.

## 1. PEROXISOME PROLIFERATOR-ACTIVATED RECEPTOR (PPAR)

PPAR and retinoid X receptor (RXR) are ligand-activated transcription factors of the nuclear hormone receptor superfamily [1, 2]. PPAR exists as 3 isoforms (*α*, *γ*, and *δ*/*β*) with distinct natural agonists for each isoform. RXR also exists as 3 isoforms (*α*, *β*, and *γ*), but all 3 can be activated by common ligands [3]. Both RXR and PPAR are composed of a ligand binding domain (LBD) and a DNAbinding domain (DBD) [4]. When their respective ligands bind to PPAR and RXR, they form a heterodimeric complex which recruits other coactivators including PPAR coactivator-1 and -2, PPAR-binding protein, PPAR-interacting protein, CREB-binding protein and steroid receptor coactivator-1. This complex binds to the promoter regions of specific genes that contain a regulatory element known as the peroxisome proliferator response element (PPRE; AGGTCA-AGGTCA repeats) which either activates or transrepresses the target genes [1, 5]. Binding of a specific agonist to PPAR is a prerequisite for coactivator binding. In the absence of a ligand, the PPAR*γ*:RXR complex can recruit corepressor complexes and bind to PPRE, suppressing the transcription of target genes [6]. Thus PPARs can control the gene expression positively as well as negatively.

## 2. FUNCTIONAL SIGNIFICANCE OF PPARS 

In the mammalian body, PPARs control glucose and lipid metabolism, cell proliferation and differentiation [1, 7]. In particular, the PPAR*γ* isoform behaves as a “molecular sensor”, binding a wide range of molecules involved in metabolism, and has been studied extensively in diabetes and obesity due to its role in regulating glucose metabolism [8, 9]. A class of synthetic, insulin-sensitizing compounds called thiazolidinediones (TZDs) have emerged as potent, exogenous agonists of PPAR*γ* and are being prescribed for type-2 diabetes [1, 10]. PPAR*γ* shows a highly restricted pattern of expression. It is present at a high amount in adipose tissue where it regulates adipocyte differentiation and lipid metabolism [5]. Its expression is also very high in cells of the immune system such as monocytes/macrophages, B and T cells [11]. In the normal adult brain, PPAR*γ* shows a relatively low level of expression primarily limited to the granule cells of the hippocampal dentate gyrus [11]. Some PPAR*γ* expression is also present in the caudate putamen and globus pallidus of the basal ganglia, thalamus, and the piriform cortex [2]. Recent studies indicated that microglia and astrocytes, the cell types that play a significant role in the inflammatory responses of the CNS show high expression levels of PPAR*γ* [12]. More recently, several TZDs including the United States Food and Drug Administration (FDA) approved rosiglitazone and pioglitazone were shown to control inflammation in peripheral organs as well as CNS [13, 14].

## 3. PPAR LIGANDS

The endogenous agonists of PPAR*α* include eicosinoids-like leukotriene B_4_ and 8(S)-hydroxy-eicosatetraenoic acid [15]. Whereas 15-deoxy-delta-12,14-prostaglandin-J2 (15dPGJ2), and several oxidized metabolites of hydroxyl-eicosatetraenoic acid and hydroxyl-eicosadecaenoic acid are the natural ligands for PPAR*γ* [16]. Many prostanoids are the natural ligands for PPAR*δ*/*β* [17]. The 3 RXR isoforms use common ligands that include 9-cis retinoic acid, docosahexaenoic acid, and phytanic acid [3].

## 4. PROMOTERS OF STROKE-INDUCED BRAIN DAMAGE

Following stroke, while the ischemic core undergoes irreversible damage, the penumbra (tissue surrounding the core) can potentially be rescued with timely therapeutic intervention [18]. Typically, the penumbra is much larger in volume than the core to start with, but as the cell death progresses with time, the infarct grows in size engulfing penumbra [19]. The secondary neuronal death that eventually precipitates the long-term neurological dysfunction after stroke is caused by many synergistic pathophysiological mechanisms involving various cell types. In particular, massive inflammation that starts immediately and continues for days after focal ischemia is a major promoter of ischemic neuronal death [20]. In core of injury, anoxic depolarization promotes calcium and potassium release leading to neurotransmitter glutamate release. This follows with a wave of spreading depression which promotes further glutamate release in penumbra. Increased extracellular glutamate promotes excitotoxic secondary neuronal death in core as well as penumbra. Immediately after stroke, due to lack of oxygen and glucose, the ionic gradients across cell membranes collapse leading to water influx and edema in CNS. In addition, mitochondrial failure leads to endoplasmic reticulum (ER) stress and oxidative stress. This is followed by the increased expression of inflammatory genes and infiltration of leukocytes into brain parenchyma. All these pathophysiological events are thought to synergistically promote the postischemic neuronal death [21].

## 5. INFLAMMATION AFTER STROKE

In a normal brain, the blood-brain barrier (BBB) controls the infiltration of white blood cells into brain parenchyma. However, following ischemia induction of the adhesion molecules like intercellular adhesion molecule-1 (ICAM1), E-selectin, and P-selectin on the endothelial cells promotes leukocyte adherence and extravasation [20]. The infiltrated macrophages and neutrophils activate resident microglia and astrocytes [22]. Following stroke, leukocytes as well as neurons, astrocytes, microglia, and oligodendrocytes generate proinflammatory mediators including cytokines like interleukin (IL)-6 and IL-1*β*, chemokines like macrophage inflammatory protein-1*α* and monocyte chemoattractant protein-1 (MCP1), prostaglandins and free radicals which exacerbate postischemic secondary neuronal death [23, 24].

## 6. ROLE OF TRANSCRIPTION FACTORS IN POSTISCHEMIC INFLAMMATION

Transcription factors play a central role in modulating inflammation by controlling the expression of cytokines, chemokines, and other inflammatory genes. Ischemia is a known stimulator of many transcription factors including hypoxia inducible factor-1 (HIF1), signal transducer and activator of transcription-3 (STAT3), early growth response-1 (Egr1), nuclear factor (erythroid-derived 2)-like 2 (Nrf2), interferon regulatory factor-1 (IRF1), activating transcription factor-3 (ATF3), cAMP response element binding protein (CREB), cAMP response element modulator (CREM), and nuclear factor-kappa B (NF-*κ*B) that are known to be significantly modulate the postischemic inflammatory gene expression [25–28]. While the transcription factors like STAT3, IRF1, C/EBP*β*, NF-*κ*B, ATF3, and EGR1 promote neuronal damage by inducing inflammatory genes [26–31], transcription factors like HIF1, Nrf2, PPAR*α*, PPAR*γ*, and CREBare thought to be beneficial as they curtail the expression of genes that promote either inflammation or oxidative stress [32–36]. Drugs that target transcription factors could be effective as they act upstream to gene expression, thus curtailing the inflammation and other destructive pathways.

## 7. ANTI-INFLAMMATORY EFFECTS OF PPAR*γ* ACTIVATION IN THE PERIPHERAL ORGANS

Many recent studies demonstrated that PPAR*γ* agonists exert significant protection in various animal models of neurological and cardiovascular disorders [37–40]. Activated macrophages release proinflammatory cytokines such as tumor necrosis factor-*α* (TNF*α*) and IL6 and free radicals such as nitric oxide (NO) and superoxide. PPAR*γ* activation by its agonists was shown to inhibit the expression of inducible NO synthase (iNOS) and inflammatory cytokine production in macrophages and endothelial cells [5, 41, 42]. PPAR*γ* agonists were also shown to reduce ROSformation in coronary artery endothelial cells and cardiac fibroblasts [43, 44]. Pioglitazone was shown to curtail ICAM1 and MCP1 expression leading to decreased macrophage infiltration after cardiac ischemia leading to curtailed myocardial damage in rats [45]. PPAR*γ* natural ligand 15dPGJ2 was shown to prevent the expression of IL6, IL1*β*, and TNF*α* in phorbol 12-myristate 13-acetate-stimulated monocytes [42]. The systemic inflammation in joints of rheumatoid arthritis patients and in the pancreas of diabetics was also shown to be minimized by treatment of PPAR*γ* agonists [8, 46–48]. Another agonist of PPAR*γ*, rosiglitazone treatment was shown to limit neutrophil infiltration, nitrotyrosine formation and lipid peroxidation following experimental pancreatitis in mice [49]. Rosiglitazone was also shown to decrease matrix metalloproteinase (MMP)-9 expression, T-cell activation, and TNF*α* and amyloid-A levels in diabetic patients with coronary artery disease, thereby curtailing the inflammatory response [50]. Both pioglitazone and rosiglitazone were shown to decrease inflammation in kidney to prevent nephropathy resulting from diabetes and hypertension [51]. In experimental animals, 15dPGJ2 was shown to inhibit NF-*κ*B activation and other proinflammatory proteins like activating protein-1 (AP-1), iNOS, and ICAM1, thereby decreasing oxidative stress to protect kidneys from ischemic damage [51–53]. Upon activation, TZD pretreated peripheral blood monocytes show decreased cytokine release and altered inflammatory gene expression [42, 54]. It was shown that PPAR*γ* activation antagonizes transcription factors STAT, NF-*κ*B, and AP1 by decreasing their DNAbinding leading to decreased expression of the downstream genes iNOS, MMP9, and scavenger receptor-A [41, 55, 56].

## 8. PPAR*γ* ACTIVATION AND STROKE

Massive inflammation is a known precipitator of stroke-induced brain damage [20, 21, 57]. Many anti-inflammatory compounds including minocycline, curcumin, caffeic acid phenyl ester and Brazilein can limit cerebral inflammation, and thus ischemic neuronal death [58–61]. As brain damage following focal ischemia is known to be mediated by many synergistic mechanisms including edema, ionic imbalance, apoptosis, oxidative stress, and ER stress, combination of drugs that prevent some if not all these pathophysiological changes might be needed to efficiently control ischemic neuronal death.

Consistent with the known benefits of PPAR*γ* activation in conditions of inflammation, several animal studies have demonstrated the therapeutic potential of TZDs in improving postischemic functional outcome. As 2 TZDs rosiglitazone and pioglitazone are currently approved by FDA for type-2 diabetes treatment and as the incidence of stroke and stroke-induced brain damage are higher in type-2 diabetics, these studies assume great importance [32, 62, 63]. Following transient focal ischemia in rodents, cerebral PPAR*γ* expression significantly elevates, especially in the peri-infarct area, and treatment with PPAR*γ* agonists transrepress the expression of many downstream proinflammatory genes [64]. Furthermore, rosiglitazone or pioglitazone treatment increases PPAR*γ* translocation to the nucleus in neurons that will be enhanced by RXR agonist retinoic acid [64, 65].

Both pretreatment as well as posttreatment with TZDs was shown to induce neuroprotection after focal ischemia [32, 66–69]. In addition, TZD treatment was observed to be effective irrespective of the route of administration. Postischemic neuroprotection was observed following intraperitoneal (i.p.) injections, intracerebroventricular (i.c.v.) infusion as well as feeding animals with chow enriched with a TZD [32, 66–68]. In adult rats and mice, pretreatment with troglitazone, rosiglitazone, or pioglitazone (i.p.) prior to ischemia was shown to decrease the infarct volume, microglial activation, macrophage infiltration, and expression of proinflammatory genes cyclooxygenase-2 (COX2), iNOS, and IL-1*β* mRNA in the ischemic hemisphere [32, 66, 68]. The rosiglitazone neuroprotection was observed to be completely reversed by treating rats with GW9662 (a specific PPAR*γ* antagonist) indicating a direct involvement of PPAR*γ* [32]. Pioglitazone when infused i.c.v. for 5 days prior to and 2 days after focal ischemia significantly increased the sensory neurological scores and reduced edema and infarct volume [67]. In another study, rats tube fed with rosiglitazone for 7 days prior to and 3 days after transient focal ischemia showed increased endothelial NO synthase (eNOS) expression and neoangiogenesis leading to ischemic tolerance [70]. Pioglitazone pretreatment was not observed to be neuroprotective following permanent focal ischemia in rats, suggesting that the beneficial effects of TZDs are limited to reperfusion-induced damage [71]. Importantly, TZD-induced neuroprotection was also observed in hypertensive and diabetic rodents subjected to transient focal ischemia [32].

## 9. PPAR ACTS TOGETHER WITH OTHER TRANSCRIPTION FACTORS

The transrepression of inflammatory genes by PPAR acts in cooperation with many other transcription factors including NF-*κ*B, AP-1, Egr1, and c/EBP*β*, and by inhibiting the ubiquitylation/degradation of corepressor proteins via sumoylation [72]. Vascular inflammation was shown to be controlled by the interaction of PPAR*γ* and c/EBP*β* by negatively regulating the expression of inflammatory genes like IL-6, IL1*β*, and TNF*α* [73]. This is made possible by the presence of tandem repeats of c/EBP*β* motif in the PPAR*γ* promoter region enabling transactivation of PPAR*γ* gene. The PPAR:RXR heterodimer complex also competes with the coactivator complexes as well as interacts directly with other transcription factors to regulate their function. In addition, TZDs can have PPAR*γ*-independent actions that include mitochondrial dysfunction-related, stress-response, increased astrocyte, glucose uptake and lactate production, and modulation of the mitochondrial protein MitoNeet [74, 75].

## 10. EFFICACY OF PPAR*γ* AGONISTS AFTER FOCAL ISCHEMIA

Of the two FDA-approved TZDs, pioglitazone is known to cross BBB more efficiently than rosiglitazone, but the affinity of pioglitazone to PPAR*γ* is 10 times lower (Kd of *∼*400 nM) than for rosiglitazone (Kd of *∼*40 nM) [1, 76]. In addition to stimulating PPAR*γ*, pioglitazone also functions as a partial agonist of PPAR*α*, whereas rosiglitazone functions as a pure PPAR*γ* agonist [13]. To make things complicated, recent studies demonstrated that to induce the same degree of neuroprotection following focal ischemia or SCI, comparable doses of pioglitazone and rosiglitazone are needed [32, 77].

## 11. NONINFLAMMATION-RELATED NEUROPROTECTIVE ACTIONS OF TZDS

Although preventing inflammation seems to be the major neuroprotective mechanism of PPAR agonists after stroke, both PPAR*γ* and PPAR*α* agonists were shown to induce other beneficial effects like reducing oxidative stress, increasing endothelial relaxation, and preventing apoptosis in the postischemic brain [63, 78, 79]. When oxidative stress was induced in immortalized mouse hippocampal cells by exposing to glutamate or hydrogen peroxide, PPAR*γ* agonists protected the cells from death [39]. Transient focal ischemia is known to promote reactive oxygen species (ROS) production and reduce glutathione levels (which scavenge ROS) simultaneously [80]. This leads to enormous oxidative stress and neuronal death. The cytosolic antioxidant enzyme, endothelial copper/zinc-superoxide dismutase (Cu/Zn-SOD) is known to decrease oxygen-free radicals to mitigate eNOS inactivation [81]. Catalase, the other major antioxidant enzyme which is very active in peroxisomes of both neurons and glial cells [82, 83] protects cells by quickly degrading hydrogen peroxide. Neurons are extremely labile to oxidative damage and cellular stress is a known inducer of both Cu/Zn-SOD and catalase expression to counter the oxidative stress and to protect neurons following focal ischemia. As the promoters of SOD and catalase genes contain PPRE, they are directly upregulated when PPAR*γ* is activated [81, 84].

Recent studies from our laboratory showed that in normotensive and hypertensive rodents subjected to transient focal ischemia, rosiglitazone treatment significantly increases Cu/Zn-SOD and catalase activity in the peri-infarct region which might be responsible for the observed neuroprotection [32]. In addition, rosiglitazone also decreased COX-2 and iNOS levels (indicating reduced production of ROS and NO) in peri-infarct neurons [34]. It was also reported that both pioglitazone and rosiglitazone significantly prevent glutathione depletion following focal ischemia in adult rats [80].

As ROS promotes apoptosis and TZDs minimize ROS formation, PPAR*γ* activation was thought to prevent postischemic apoptotic neuronal death [85, 86]. A recent study showed that rosiglitazone treatment decreases caspase-3 levels leading to reduced apoptotic cell death following focal ischemia [87]. This seems to be a direct PPAR*γ* downstream effect as pretreating animals with the PPAR*γ* antagonist GW9662 prevented the antiapoptotic actions of rosiglitazone [87]. Pioglitazone treatment was also shown to promote the expression of antiapoptotic gene Bcl2 while simultaneously preventing the expression of proapoptotic gene Bax in the peri-infarct regions of brain following focal ischemia [13, 88].

## 12. PPAR*γ* AGONIST-INDUCED NEUROPROTECTION IN HUMAN STROKE SUBJECTS

A recent clinical trial, named the Prospective Pioglitazone Clinical Trialin Macrovascular events (proactive) started evaluating if pioglitazone treatment can prevent the macrovascularevents in type-2 diabetes [89, 90]. This extensive study evaluated 5, 238 patients in 19 countries. In particular, one prespecified subgroup analysis evaluated the effect of pioglitazone in patients with (*n* = 984) or without (*n* = 2867) a history of stroke and observed a 16% relative risk reduction in the pioglitazone group compared to placebo group [91]. In addition, within the group of patients with a previous stroke, pioglitazone therapy decreased the risk of recurrent stroke by 47% compared to placebo over 3 years. But pioglitazone had no effect on decreasing first strokes over this period. While this shows an encouraging trend for stroke patients, serious heart failure was observed to be increased significantly in the pioglitazone group compared to placebo group. The complete details and results of the proactive trial can be viewed at the website http://www.proactive-results.com/index.htm. Yki-Järvinen [92] commented critically on this trial that pioglitazone group showed increased edema and increased incidence of pneumonia. Furthermore, the weight gain was 4 kg greater in the pioglitazone over placebo group which is undesirable. A recent study also showed that pioglitazone or rosiglitazone therapy significantly enhanced the functional recovery in a group of 30 type-2 diabetes patients admitted in the hospital for acute stroke rehabilitation [93].

## 13. BENEFICIAL EFFECT OF PPAR*γ* ACTIVATION IN OTHER CNS INJURIES

PPAR*γ* activation by TZDs was shown to prevent inflammation and neuronal death in several in vitro and in vivo models of CNS diseases [40, 94–97]. In patients suffering with Alzheimer disease, PPAR*γ* activation was shown to prevent TNF*α* and iNOS expression in macrophages, thus limiting the inflammation and cognitive impairment [40, 98]. TZDs were known to significantly reduce the dopaminergic neuronal loss leading to improved neurological status in Parkinson’s disease [99]. Using the rodent model of multiple sclerosis (experimental autoimmune encephalomyelitis), PPAR*γ* agonist treatment was shown to suppress activation of T-cells, microglia, and macrophages thus decreasing proinflammatory factor formation leading to improved neurological outcome [94, 96, 100]. Pioglitazone oral treatment was reported to decrease the microglial activation, motor neuron loss, and muscular atrophy in transgenic mice overexpressing SOD1-G93A (an animal model of Amyotrophic lateral sclerosis) with pioglitazone [101, 102]. These mice also showed increased anti-inflammatory gene expression upon treatment with pioglitazone [101]. More recently, two studies showed the beneficial effects of treating rodents with PPAR*γ* agonists following spinal cord injury (SCI) [77, 103]. Our laboratory demonstrated that both rosiglitazone and pioglitazone decreases inflammatory cell activation and inflammatory gene expression leading to smaller lesion size, better motor recovery, and less neuropathic pain after SCI in rats [77]. Importantly, we showed that pretreating rats with the PPAR*γ* antagonist GW9662 prevents many beneficial effects of TZDs following SCI indicating a direct mediation of PPAR*γ* in promoting post-SCI neuronal recovery [77].

## 14. PPAR*γ*-INDEPENDENT NEUROPROTECTIVE ACTIONS OF PPAR*γ* AGONISTS

While the anti-inflammatory and neuroprotective actions of PPAR*γ* agonists are expected to be mediated via PPAR*γ* stimulation, some studies suggested that PPAR*γ* agonists also induce many beneficial effects via PPAR*γ*-independent mechanisms as well. 15dPGJ2 was shown to prevent astroglial and microglial activation by bacterial endotoxins without involving PPAR*γ* [12]. The anti-inflammatory action of 15dPGJ2 was shown to be mediated by binding to and inactivating inhibitor of kappa B (I*κ*B) kinase and by alkylating the p50/p65 dimers and thus preventing the activation of NF-*κ*B without involving PPAR*γ* [52, 55]. Following focal ischemia, the proinflammatory actions of the cytokine IL6 are known to be mediated by the activation of IL6 receptor-associated Janus kinases (JAKs) and their downstream STAT family of transcription factors [25]. Suppressor of cytokine signalling (SOCS) proteins act as negative feedback regulators and inhibit JAK and STAT phosphorylation, thus preventing the upregulation and binding of cytokines to their receptors after an acute CNS insult [32, 104]. Recent studies from our group and others demonstrated that rosiglitazone and 15dPGJ2 induce SOCS3 expression and prevent JAK2 and STAT3 phosphorylation [32, 105]. Thus the neuroprotective actions of PPAR*γ* agonists might also mediated by direct actions on JAK-STAT-SOCS pathway. Our recent studies also showed that pioglitazone treatment after SCI induces the heat shock protein (HSP)-27, HSP70, and HSP32 (hemeoxygenase-1) which induce neuroprotection [104].

## 15. ACTIVATION OF OTHER PPAR ISOFORMS ALSO INDUCES NEUROPROTECTION

In addition to PPAR*γ*, PPAR*α*, and PPAR*β*/*δ*, activation was also shown to significantly prevent inflammation and induce protection after injury to CNS as well as peripheral organs. PPAR*α* activation is known to induce neuroprotection after focal ischemia [36, 78]. PPAR*α* plays a very important regulatory role in response to injury or stress. As PPAR*α* is known to be expressed when monocytes differentiate into macrophages, it influences postinjury inflammatory reactions [105]. Furthermore, agonist-induced activation of PPAR*α* increases I*κ*B*α* levels leading to an inhibition of NF-*κ*B [106]. PPAR*α* activation decreases the level of proatherosclerotic fibrinogen and C-reactive protein in experimental animals [107]. Fenofibrate, a potent exogenous agonist of PPAR*α*, inhibits left ventricular hypertrophy by stimulating free fatty acid uptake and *β*-oxidation [63]. Fenofibrate pretreatment reduces the susceptibility of mice deficient in apolipoprotein-E, and decreases the infarct volume in wild type mice subjected to focal cerebral ischemia [78]. Poststroke neuroprotection induced by PPAR*α* agonists will be mediated by both cerebral and vascular mechanisms. Fenofibrate treatment is known to decrease vascular endothelial dysfunction and improves endothelium-dependent vasodilatation in patients with hypertriglyceridemia [91]. Recent studies showed that a PPAR*α*/*γ* dual agonist bezafibrate decreases anaerobic metabolism and there by prevents death in gerbils subjected to global cerebral ischemia [108]. Fibrates are also reported to prevent secondary neuronal death by oxidative stress by enhancing the expression of antioxidant enzyme, Cu/Zn-SOD, and by decreasing vascular cell adhesion molecule-1 expression in CNS blood vessels, possibly by inhibiting the NF-*κ*B pathway [98, 109]. The functional significance of PPAR*β*/*δ* in preventing CNS inflammation is not studied in detail. However, the PPAR*β*/*δ* agonists L-165041, and GW501516 were shown to significantly decrease focal ischemia-induced infarction and brain damage in adult rats [110]. In rodents, PPAR*β*/*δ* agonists were also demonstrated to prevent striatal dopamine loss after 1-methyl 4-phenyl 1,2,3,6-tetrahydropyridine administration [110]. PPAR*β*/*δ* agonist GW0742 was reported to inhibit lipopolysaccharide-induced TNF*α* secretion in cardiomyocytes [111].

## 16. CONCLUSIONS

Despite decades of research, no therapies that can prevent the secondary neuronal death and the ensuing neurological deficits after stroke are currently available. Many pathological mechanisms including inflammation, ionic imbalance, excitotoxicity, edema, oxidative stress, and ER stress synergistically promote the poststroke secondary neuronal death. Hence therapeutics that simultaneously targets several of these mechanisms with minimal side effects is extremely useful in stroke therapy. PPAR*γ* agonists like rosiglitazone and pioglitazone are FDA approved and being prescribed to millions of type-2 diabetics allover the world. The benefit of these compounds seems to be their potential to influence multiple molecular mechanisms. For example, they are known to minimize the harmful events like inflammation and oxidative stress at the same time promote the antioxidant defence and protein chaperones. Hence PPAR*γ* agonists might be an important class of drugs for use in stroke therapy. The benefits of PPAR*γ* agonist treatment was also observed in other acute CNS injuries like SCIas well as chronic neurodegenerative disorders like multiple sclerosis, Alzheimer’s disease, and Parkinson’s disease increasing the promise of these compounds as future neuroprotective therapies.
